# Maximising retention in a longitudinal study of genital *Chlamydia trachomatis *among young women in Australia

**DOI:** 10.1186/1471-2458-11-156

**Published:** 2011-03-09

**Authors:** Jennifer Walker, Christopher K Fairley, Eve Urban, Marcus Y Chen, Catriona Bradshaw, Sandra M Walker, Basil Donovan, Sepehr N Tabrizi, Kathleen McNamee, Marian Currie, Marie Pirotta, John Kaldor, Lyle C Gurrin, Hudson Birden, Veerakathy Harindra, Francis J Bowden, Suzanne Garland, Jane M Gunn, Jane S Hocking

**Affiliations:** 1 Centre for Women's Health, Gender and Society, School of Population Health, University of Melbourne, Victoria 3010, Australia; 2 Sexual Health Unit, School of Population Health, University of Melbourne, and Melbourne Sexual Health Centre, Melbourne Victoria 3010, Australia; 3 Sexual Health Unit, School of Population Health, University of Melbourne, Victoria 3010, Australia; 4 Department of Epidemiology and Preventive Medicine, Monash University, and Melbourne Sexual Health Centre, Melbourne Victoria 3010, Australia; 5 National Centre in HIV Epidemiology and Clinical Research, University of NSW, Sydney 2000, Australia; 6 Department of Molecular Microbiology, The Royal Women's Hospital, Melbourne, Victoria, Australia; 7 Family Planning Victoria, Melbourne, Australia and Monash University, Victoria, Australia; 8 Australian National University, Canberra, Australia; 9 Primary Care Research Unit, Department of General Practice, University of Melbourne, Victoria 3010, Australia; 10 Centre for Molecular, Environmental, Genetic and Analytic Epidemiology, School of Population Health, University of Melbourne, Victoria 3010, Australia; 11 North Coast Medical Education Collaboration, Sydney School of Public Health, University of Sydney, Lismore, NSW, Australia; 12 St Mary's Hospital, Portsmouth, UK; 13 The Royal Women's Hospital, Melbourne, Victoria, Australia; and the Department of Obstetrics and Gynaecology, The University of Melbourne, Victoria, Australia

## Abstract

**Background:**

Cohort studies are an important study design however they are difficult to implement, often suffer from poor retention, low participation and bias. The aims of this paper are to describe the methods used to recruit and retain young women in a longitudinal study and to explore factors associated with loss to follow up.

**Methods:**

The Chlamydia Incidence and Re-infection Rates Study (CIRIS) was a longitudinal study of Australian women aged 16 to 25 years recruited from primary health care clinics. They were followed up via the post at three-monthly intervals and required to return questionnaires and self collected vaginal swabs for chlamydia testing. The protocol was designed to maximise retention in the study and included using recruiting staff independent of the clinic staff, recruiting in private, regular communication with study staff, making the follow up as straightforward as possible and providing incentives and small gifts to engender good will.

**Results:**

The study recruited 66% of eligible women. Despite the nature of the study (sexual health) and the mobility of the women (35% moved address at least once), 79% of the women completed the final stage of the study after 12 months. Loss to follow up bias was associated with lower education level [adjusted hazard ratio (AHR): 0.7 (95% Confidence Interval (CI): 0.5, 1.0)], recruitment from a sexual health centre as opposed to a general practice clinic [AHR: 1.6 (95% CI: 1.0, 2.7)] and previously testing positive for chlamydia [AHR: 0.8 (95% CI: 0.5, 1.0)]. No other factors such as age, numbers of sexual partners were associated with loss to follow up.

**Conclusions:**

The methods used were considered effective for recruiting and retaining women in the study. Further research is needed to improve participation from less well-educated women.

## Background

Cohort studies are one of the most important study designs in modern epidemiology. While they can be complex to organize and expensive to conduct, they have a considerable advantage over case control studies in that they avoid several important sources of bias which might be introduced by the participants when they know their disease status, by the researchers when they know whether a participant is a case or control, and in the selection of the controls[1]. However, the validity of the results of a cohort study can be severely compromised if participation is low, there is substantial bias, or if there is significant loss to follow up of study participants, particularly if this loss to follow up is related to their exposure. Representative samples of young people can be particularly difficult to recruit and retain in a longitudinal study, in part because of the difficulty in identifying an appropriate sampling frame and also because young people change address frequently[2]. Other published longitudinal studies of young women have been limited by high loss to follow up, low participation rates and retention bias[2-5].

We have recently completed a 12 month longitudinal study of young Australian women aged 16 to 25 years - the Chlamydia Incidence and Re-Infection Rates Study (CIRIS). This study recruited women from primary care clinics and aimed to measure the incidence of and risk factors for, genital *Chlamydia trachomatis *(chlamydia) infection [results presented elsewhere][6].

The aims of this paper are to detail the methods used to recruit and retain a representative sample of women in a longitudinal study. We also describe the population of women recruited and explore factors associated with loss to follow up, in order to provide other researchers with ideas for maximizing participation in future longitudinal studies.

## Methods

### Participants

CIRIS was a prospective cohort study of 16 to 25 year old Australian women recruited through general practice (GP) clinics and family planning/sexual health clinics. Women were eligible for the study if they had ever been sexually active, were not knowingly pregnant at the time, were competent with English as a written language and were able to be followed up using the Australian postal system during the following 12 month period.

### Sampling frame and recruitment method

The sampling frame for this study was primary care clinics in the states of Victoria, New South Wales and the Australian Capital Territory. Between May 2007 and August 2008, eligible female patients were recruited from 29 separate GP and family planning/sexual health clinics in rural and urban areas in south eastern Australia, where 60% of the population of Australia live[7]. To facilitate obtaining a reasonably representative sample, clinics were chosen as recruitment sites on the basis of the socio-demographic profile of their local area[7]. Research assistants were based in each clinic for up to six weeks, and approached all 16 to 25 year old women presenting for a consultation, irrespective of the reason for their presentation, to invite them to participate in the study. All research assistants were female and were chosen to fit in with the type of clinic they were recruiting from: for example younger research assistants recruited in youth clinics and mature aged research assistants recruited from general practice. To ensure minimal disruption to the clinic clients and staff, all research assistants were trained and supervised closely, and the CIRIS research team liaised frequently with clinic personnel. Confidentiality and discretion were maintained by discussing the study with patients in a private space within the clinic, and information provided to the research team was not disclosed to their clinician unless requested by the participant. Research assistants were independently employed by the University of Melbourne to minimise any impact on the clinic and to separate the relationship between the study and the participant's usual clinical care.

Once recruited, all participants received a study pack with a copy of their consent form, the plain language statement, information about the project, and information on chlamydia and other sexually transmissible infections (STI). In addition, all participants were given condoms and lubricant.

At the time of recruitment (referred to as 'baseline'), the participants completed a questionnaire which collected demographic information, sexual behaviour data (number of opposite and same sex partners and condom use), recent antibiotic and contraceptive use data, and the presence of any genital symptoms. Questionnaires were sent out at each three month follow up requesting information relevant to the previous three months including numbers of new sexual partners, contraceptive use, details about recent GP visits, STI testing, antibiotic use, and any pregnancies, including miscarriage or termination of pregnancy.

### Follow up

All participants were asked to self-collect a vaginal swab at baseline, six and 12 months for chlamydia testing and anyone who tested positive for chlamydia at baseline or at six months, was also required to return a swab three months later as a test for re-infection. Participants were also asked to complete a sexual behaviour questionnaire every three months regardless of whether or not they collected a swab. All follow up was done through the standard Australian postal service, and follow up packs were designed to be nondescript, simple to use and free. A prompt was sent via SMS (mobile phone text message) or email a week prior to sending out the follow up kit (depending on the participant's preference) to alert the participants to expect a delivery, and this was also a cue for them to update their contact details if necessary. Similarly, a reminder was sent if the follow up pack had not been returned within two weeks. If there was still no response after a further two weeks, research staff made up to ten telephone calls at different times of the day and different days of the week. For continuity, the same staff followed up the participants throughout the study. Also, the participants were able to contact study staff at any stage by calling a free-call telephone number or emailing the CIRIS email address. A website was designed with detailed information about the study and participants could also notify any changes in their contact details via the website. Participants were excluded from further follow up if their telephone number was disconnected/continually unanswered, emails 'bounced back', or their follow up parcels were 'returned to sender'.

Incentives were provided in the form of gift vouchers redeemable at a number of large retail outlets; at three months, a AUD$10 voucher, AUD$20 at six and nine months and AUD$50 at 12 months. Small gifts (eg tampons, confectionery, cosmetics) were included in all follow up kits if this had been agreed to by the local ethics committee.

### Generalisability and bias

To assess the generalisability of the study, the demographics and sexual behaviour profiles reported at baseline were compared with the general population of women in Australia in the same age group.

To determine any selection bias, women who declined to participate were compared with participants; however due to ethical considerations, the only information available was the age of the women and the clinic they attended. The participation rate was calculated by determining the number of women who were recruited relative to the number of eligible female patients attending that service. Where possible, the age and reason for refusal was collected for all non-participators.

To identify any loss to follow up bias, the demographics and sexual behaviour information, and chlamydia test results of the participants who had completed the follow up at the time of their final response, were compared with the participants who were lost to follow up. Reasons for withdrawing or not completing the study were not always forthcoming however when available, these were included in the results.

### Sample size

Assuming a design effect of 2, a sample size of 860 was required to obtain a chlamydia incidence of 4.5% per year (± 2.0), as chlamydia incidence was the primary aim of the CIRIS study. We assumed a 20% loss to follow up and aimed to recruit a sample of 1100 women.

### Statistical analysis

For the purposes of this analysis, participants were regarded as 'lost to follow up' if they did not provide a swab and complete a questionnaire for the final stage of the study. All data were analyzed using STATA version 10.2[8]. All analyses were adjusted for clustering at the individual clinic level. Hazard ratios and adjusted hazard ratios and robust standard errors were calculated using Cox regression methods to explore associations between women who remained in the study and participants who were lost to follow up. Each individual's observation period was represented in the dataset thus allowing variables such as number of new sex partners to be recorded separately for each time period. Age was categorized as 16 to 20 years versus 21 to 25 years for some of the analyses.

Ethics approval to conduct this study was obtained from ten Human Research Ethics Committees throughout Australia including: The University of Melbourne Health Sciences Human Ethics Sub-Committee, Bayside Health Service District Human Research Ethics Committee, ACT Health and Community Care Human Research Ethics Committee, Family Planning Victoria Ethics Committee, North Coast Area Health Service Human Research Ethics Committee, South Eastern Sydney and Illawarra Area Health Service Human Research Ethics Committee, The University of Newcastle Human Research Ethics Committee, University of NSW Human Research Ethics Committee, The University of Ballarat Human Research Ethics Committee, and the Family Planning NSW Ethics Committee.

## Results

### Participants

Recruiting staff discussed the study with 2835 consecutive 16 to 25 year old female patients from 29 clinics. Of these women, 1137 were ineligible for participation; 297 (26%) had never had vaginal sex with a man, 341 (30%) were travelling or otherwise unable to receive mail, 47 (4%) were not competent to consent or were not literate in English, 114 (10%) were pregnant at the time of recruitment, and 338 (30%) for other reasons. Of the 1698 eligible women, 582 (34%) declined to be in the study, 452 (78%) who declined were 'not interested', 105 (18%) stated they had 'no time', and 19 (3%) of women declined for other reasons.

**Figure 1:**
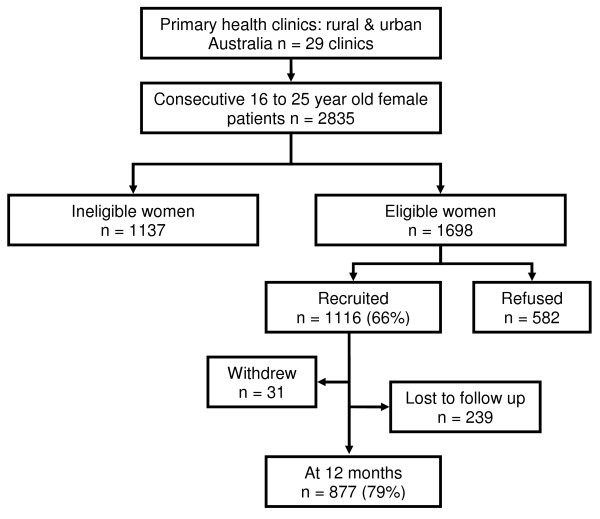
**Flowchart representing recruiting procedure including number of women who were ineligible, refused and consented to the CIRIS study**.

Of the 1698 eligible women, 1116 consented to the study, giving a participation rate of 66% (Figure 1). There was no difference in participation by age of women (21 to 25 years compared with 16 to 20 years) (OR: 1.1, 95% CI: 0.8, 1.5).

The median age of participants was 21 years and 738 (66%) were recruited from GP clinics. Participants were more likely to be Australian born and more well-educated in comparison with the underlying Australian population of the same age (Table 1)[7]. Participants were also more sexually active on average in comparison with a representative sample of Australian women reported in the 2001 national sexual behavior study (Table 1)[9].

**Table 1:** Characteristics of the participants in the CIRIS study compared with the background Australian population of 16 to 25 year old women.

Variable	Study sample (95% CI^1^)	Background Australian population of 16 to 25 year old women
COB [7]		
Not Aust born	11.5 (9.6, 13.5)	21.6
Aust born	88.5 (86.5, 90.4)	78.4

Indigenous status [7]		
Not indigenous	97.7 (96.6, 98.5)	97.9
Indigenous	2.3 (1.5, 3.4)	2.1**^2^**

Education[7]		
Up to year 12	56.1 (53.1, 59.1)	79.1
Tertiary	43.9 (40.9, 46.9)	20.9

Employment[7]		
Unemployed/Not working	38.5 (35.6, 41.5)	40.4
Employed	61.5 (58.5, 64.4)	59.6

Number of. sexual partners 12 months prior to baseline[9]		
0 - 2	67.3 (64.3, 70.1)	95
3 - 4	19.6 (17.3, 22.2)	6.5
5+	13.1 (11.1, 15.3)	3.0

^1 ^95% confidence interval, **^2^**South Eastern Australia.

### Retention

**Figure 2:**
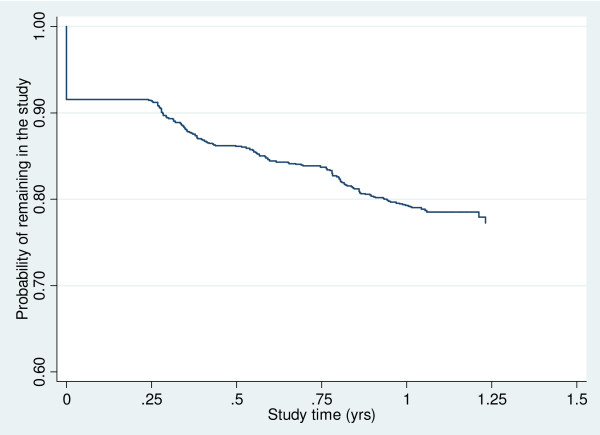
**Time under observation until loss to follow up**.

Of the 1116 participants who commenced the study, 877 (79%) completed the final stage of the study at 12 months. The largest loss to attrition was during the first three months with 94 (8%) failing to return after their initial contact (Figure 2). A total of 928 (83%) participants completed the three month follow up, 889 (80%) sent back their six month follow up, 853 (76%) returned the nine month follow up, and 877 (79%) returned the 12 month follow up. Not all participants who completed the 12 month follow up returned all study material, 392 (35%) participants skipped at least one of the three month follow ups. Only 31 participants (2.7%) withdrew from the study, with most providing no reason for withdrawal.

Participants who completed the final stage of the study were compared with those participants who were lost to follow up to assess if there was any retention bias. The crude and adjusted hazard ratios for factors associated with loss to follow up are shown in Table 2. Loss to follow up in the study was associated with being recruited from a sexual health clinic relative to a general practice [adjusted hazard ratio (AHR): 1.6 (95% CI: 1.0, 2.7)]. Loss to follow up was less likely to be associated with a past history of having chlamydia [AHR: 0.8 (95% CI: 0.5, 1.0)] or having a higher educational level [AHR: 0.7 (95% CI: 0.5, 1.0)], but no other associations were found (Table 2). The median number of new sexual partners for women retained in the study was 1.2, and for women lost to follow up was 1.7 (p = 0.4).

**Table 2:** Unadjusted and adjusted hazard ratios for women lost to follow up in the study.

Characteristic	Total number of women (n)	Number (%) lost to follow up	Hazard Ratios (95% CI)^a^	Adjusted Hazard Ratios ^b ^(95% CI)
Clinic type				
GP^c^	738	140 (19.0)	1	**1**
SHS^d^	378	99 (26.2)	1.5 (0.9, 2.4)	**1.6 (1.0, 2.7)**

Clinic location				
Rural	455	88 (19.3)	1	1
Metro	661	151 (22.8)	1.2 (0.7, 2.2)	1.4 (0.8, 2.4)

Age group				
< 21 years old	452	100 (22.1)	1	1
> 20 years old	664	139 (20.9)	0.9 (0.6,1.3)	1.0 (0.7, 1.3)

Education level achieved				
Up to year 12	609	140 (23.0)	1	**1**
Tertiary	477	85 (17.8)	0.7 (0.5, 1.0)	**0.7 (0.5, 1.0)**

Employment status				
Unemployed/Not working	418	81 (19.4)	1	1
Employed	668	143 (21.4)	1.1 (0.8, 1.5)	1.1 (0.9, 1.5)

Tested positive for chlamydia *prior *to study				
No	965	202 (20.9)	1	**1**
Yes	114	20 (17.5)	0.8 (0.6, 1.0)	**0.8 (0.5, 1.0)**

Number of new sexual partners during the study^e^				
0 - 2 partners	706	173 (24.5)	1	1
> 2 partners	410	66 (16.1)	0.8 (0.6, 1.0)	0.8 (0.5, 1.2)

^a ^95% confidence interval.

^b^Adjusted Hazard Ratios = adjusted for type of clinic recruited from, location of recruitment site, age, education level reached, employment status, numbers of new sexual partners at each stage, if tested positive prior to the study.

^c ^General practice clinic.

^d ^Sexual health service/Family Planning clinic.

^e ^Cumulative number of new partners throughout the study.

During the follow up period, 392 (35%) participants changed their postal address. Of these, 287 participants changed their address once, 87 changed their address twice, 16 changed their address three times and two changed address four times. It was not recorded how often email or telephone numbers changed, however this was commonly done.

## Discussion

This study was successful in retaining a high proportion (nearly 80%) of participants with minimal attrition bias. Considering the study required participants to complete questionnaires about sensitive subject matter and provide self-collected genital samples through the post, we would suggest that the methods we used may assist others planning similar studies.

There were limitations to the study. Australian-born women were overrepresented, not unexpectedly, considering that women with insufficient English skills were excluded from the study for logistical reasons. Participants also tended to be more highly educated compared with the background population of the same age,[7] a common finding in similar studies[9-11]. Further, women participating in this study were more sexually active on average compared with the national sexual behavior study, The 'Australian Study of Health and Relationships',[9] however, this study was conducted in 2001 and more recent data suggest young women are becoming more sexually active[12].

Our study had a number of strengths. Firstly we achieved a high retention rate of nearly 80% and a high participation rate of 66%. Secondly, a high proportion of women were recruited from general practice (66%), thus providing a more community based sample, particularly given that nearly 90% of women in this age group attend a general practitioner for their own health each year[13]. Further, our cohort had a strong representation from younger women, with nearly 50% of participants being aged less than 20 years, an age group frequently under-represented in sexual health studies[9,10].

As we wanted to estimate population prevalence and incidence, it was important that our sample was recruited using robust population sampling methods; given this, our recruitment rate of 66% was relatively high in comparison with a recent UK chlamydia incidence study [3] that reported a recruitment rate of 26% for women in primary care clinics. The Australian Longitudinal Women's Health Study, a cohort of women that aimed to measure women's health indicators reported a recruitment rate of 42% using postal questionnaires[2]. We also had an almost 80% retention of women over 12 months, which is higher than the 61% reported in the UK chlamydia incidence study of young women over an 18 month period[3]. The recently published cohort of women in Norway reported a high retention of 93% at 12 months, but this study relied on face to face meetings, which was not practical nor cost effective in the Australian context, given the vast distances between study participants[14]. Neither the UK nor the Norwegian study however, explored any potential role of retention bias.

We used face to face contact for recruitment as telephone-based and mail-based recruitment have been less effective for recruiting women into other similar studies in Australia[2,10]. Our follow up was conducted via mail, which has been demonstrated to be an acceptable method for follow up chlamydia testing and is more practical given that some participants live in rural and isolated areas[15]. However, the high retention of participants in other longitudinal studies where follow up was conducted in person either by using consultations or home visits [14,16] suggests this might be more a more effective method for retaining people in longitudinal studies, but this is expensive to implement and was not logistically practical in our case.

Overall, women who remained in the study were more well-educated, a very common finding in similar types of research[9-11]. Retention bias has been reported in other sexual health cohort studies, where loss to follow up was highest in women from low socio-economic backgrounds,[5] women recruited from sexual health centres as opposed to general practice clinics, and women who were more sexually active[4].

We would suggest that it is likely that women chose to remain in our study for a number of reasons: our participants had easy access to study staff via email or a free-call telephone number; we had regular communication with our participants; wherever possible, each participant was contacted by the same research assistant and this engendered familiarity and trust. All research assistants employed on the study were thoroughly trained and fully informed, and the research team liaised closely with clinics addressing any queries or concerns quickly. Recruiting in privacy, ensuring confidentiality, supplying appropriate information and managing positive results efficiently, respectfully and at no cost also increased trust. Further, incentive payments and the small gifts encouraged good will among participants. We also used research assistants who were independent of the clinical relationship to recruit the women (Table 3). This method was intentionally used to minimize any influence the clinical situation had on participation and to reduce the impact on the clinicians who are already very busy and may not want to discuss sexual activity unless it is considered relevant to the consultation[17].

**Table 3:** Methods utilized to increase recruiting and follow up of the participants in the study.

	**Recommended recruiting methods**:
1	Ensure all research assistants are adequately trained, fully informed and supervised closely

2	Liaise closely with clinic staff (administrative and clinical) and ensure they are fully informed about the recruiting process; set up a process to address any queries or concerns expediently

3	Recruit in privacy, ensuring the participants feel comfortable discussing the study and feel confidentiality is assured

4	Supply appropriate information about the study in plain folders

5	Employ research assistants who are independent of the clinical relationship to remain clear of the patient doctor relationship.

	**Methods to maximize retention**:

1	Communicate regularly with participants, being particularly sensitive to maintain confidentiality

2	Prompt participants prior to sending follow up to confirm contact details

3	Maintain contact with participants using the same staff to engender familiarity and trust

4	Provide easy access to study staff by email or a free-call telephone number, including being available after hours (until 9 pm weeknights and on weekends)

5	Manage positive results efficiently, respectfully and at no cost to the participant
6	Provide small incentive payments and small gifts when appropriate.

While our assessment of the effectiveness of the individual methods used has been subjective, there has been some evaluation and discussion in the literature about methods that are more likely to increase recruiting and retention in sexual health research. Strategies such as prompting participants to confirm their contact details prior to sending out follow up have been demonstrated to increase the return of postal surveys,[18] Other studies also suggest that having a dedicated research team and being flexible and creative help to increase recruitment rates [19,20] and interestingly, whilst gifts and money provide incentives to be involved, young women are only likely to be part of a study if they feel it is an altruistic thing to do[20,21].

## Conclusion

The challenges identified in this study included the nature of the research (sexual health), the required follow up (sending vaginal swabs through the post) and the mobility of the participants in the study (at least 35% moved one or more times during the 12 months). However, our methodology was very successful in terms of retention and recruitment, both of which are crucial to the success and validity of a cohort study. Further, our methods resulted in negligible retention bias, also crucial in terms of the usefulness of the study results although further research is needed to improve participation from less well-educated women. Even though we were unable to test our methodology using a randomized design, other researchers may benefit from adopting some of our methods and clearly more evaluation of effective methods is warranted.

## Competing interests

The authors declare that they have no competing interests.

## Authors' contributions

JW, managed and implemented the study, completed the analysis and led the writing; JH, was the principal investigator for the study, led analysis and conceived the study; CKF, BD, JKK, VK, FB, SG, JG, MYC, CSB, SG, KM, MP designed the study methodology; LG, was involved in the analysis; MYC, CSB, KM, were involved in the recruitment strategy and medical management of the participants, particularly in Victoria; SNT, & SG managed, designed and implemented all microbial testing; BD, JKK, HB were involved in the recruiting of participants in New South Wales; MC and FB were involved in recruiting in the Australian Capital Territory; EU, & SW, contributed to the implementation and completion of the study; all authors were involved in writing and editing this article.

## Pre-publication history

The pre-publication history for this paper can be accessed here:

http://www.biomedcentral.com/1471-2458/11/156/prepub
